# Frontiers in mechanobiology and mechanomedicine

**DOI:** 10.1007/s44258-025-00071-5

**Published:** 2025-11-28

**Authors:** Priscilla Y. Hwang, Panagiotis Mistriotis, Haogang Cai, Longwei Liu, Bo Zhang, Quinton Smith, Jacopo Ferruzzi, Peter Yingxiao Wang, Song Li

**Affiliations:** 1 Department of Biomedical Engineering, Virginia Commonwealth University, Richmond, VA USA; 2 Department of Chemical Engineering, Auburn University, Auburn, AL USA; 3 Tech4Health Institute and Department of Radiology, NYU Langone Health, New York, NY USA; 4 Department of Biomedical Engineering, New York University, Brooklyn, United States; 5 Alfred E. Mann Department of Biomedical Engineering, University of Southern California, Los Angeles, CA USA; 6 Department of Bioengineering and Medicine, University of California at Los Angeles, Los Angeles, CA USA; 7 Department of Chemical and Biomolecular Engineering, University of California at Irvine, Irvine, CA USA; 8 Department of Bioengineering, University of Texas at Dallas, Richardson, TX USA; 9 Department of Biomedical Engineering, University of Texas Southesten Medical Center, Dallas, TX USA

**Keywords:** Mechanomedicine, Immunoengineering, Cell engineering, Cell migration, Cell metabolism, Nanopatterning

## Abstract

This perspective derives from the presentations and discussions on mechanobiology at the 2025 Cellular and Molecular Bioengineering Conference in San Diego. Mechanobiological processes play critical roles in tissue development, regeneration, and disease progression. Recent advances in engineering, biology, and medicine have enabled the translation of mechanobiology discoveries into clinical practice, giving rise to the emerging field of mechanomedicine. The development and application of engineering technology and tools have provided new insights into how mechanical cues regulate immune cell response, stem cell differentiation, cell migration, and cell metabolism. In this perspective, we highlight exciting discoveries and innovative tools in mechanobiology research, and discuss challenges that must be overcome to truly bridge the gap between mechanobiology and mechanomedicine.

**Graphical Abstract:**

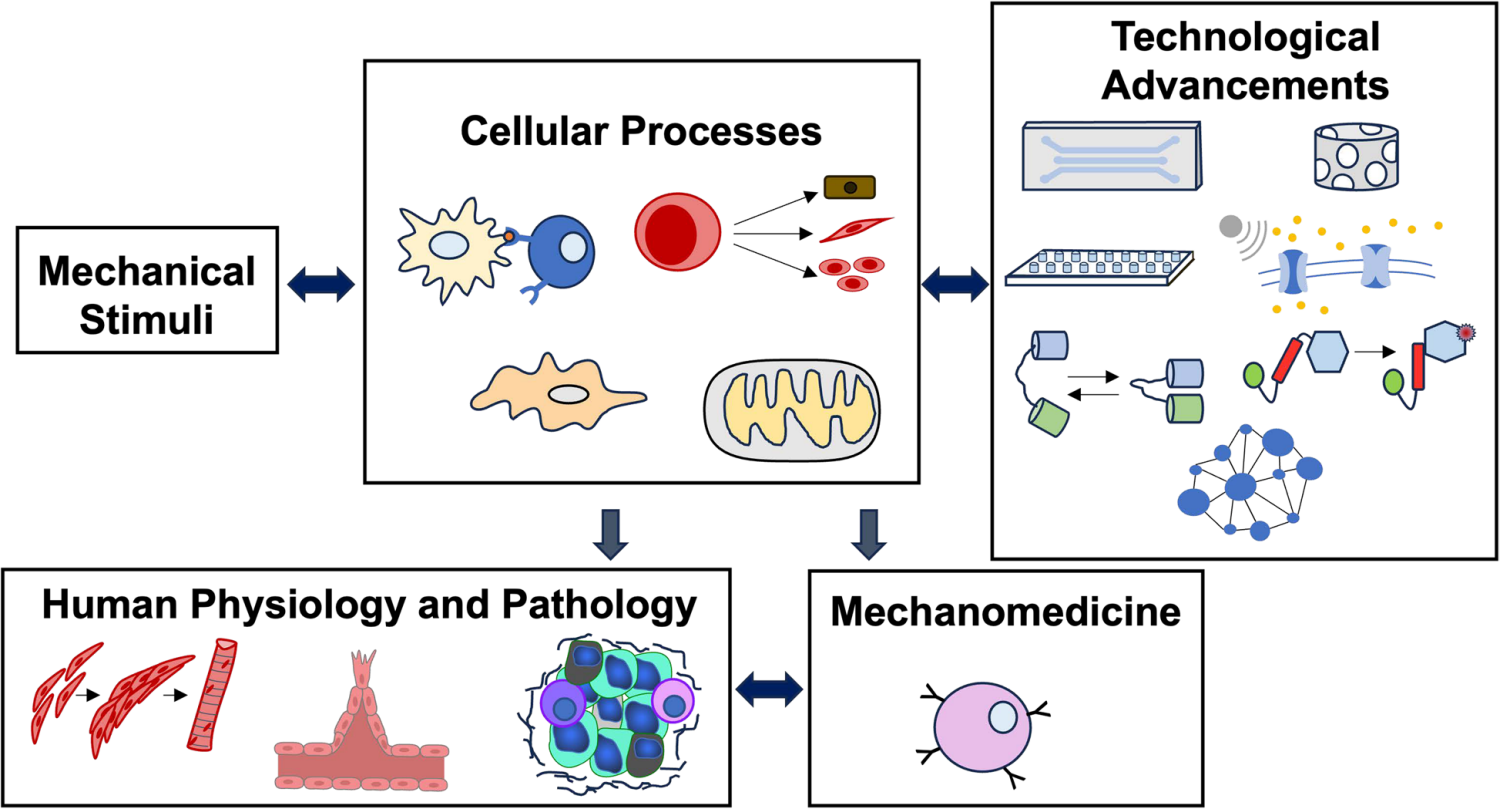

## Highlights

- In our Perspectives article, we cover recent mechanobiology advances in immune-engineering, aging, stem cells, cell migration and metabolism.
- We highlight how biomedical engineering approaches to understanding and controlling mechanical regulation of cells is leading to new clinical advances with respect to detection, diagnosis, prognosis, or treatment.
- We discuss future directions to bridge the gap between these two fields.
- We make a call to the community to build on the momentum gained in the last two–three years to further advance the fields of mechanobiology and mechanomedicine.

## Introduction

Cells are dynamic in the sense that they are a function of their intrinsic characteristics and interactions with their local environment. Within the local environment, there are many different dynamic stimuli; one category of stimuli are the mechanical aspects of the environment [1, 2]. The mechanical environment, often termed the mechano-niche, encompasses mechanical hallmarks including mechanical stress, interstitial fluid flow, and matrix properties (i.e. stiffness, architecture, pore size, etc.) [3]. Many studies have investigated how cells sense, respond, and adapt to these mechanical stimuli [4]. Discoveries in mechanobiology have informed our understanding of hierarchical mechanical regulations at the tissue, cell, sub-cellular levels, and interactions amongst these different levels. Fundamental cellular level behaviors, such as immune cell response, stem cell differentiation, cell migration and metabolism, and tissue development are dependent on mechanobiological processes. When mechanobiological processes go awry, this can lead to disease progression, such as cancer.

**Fig. 1:**
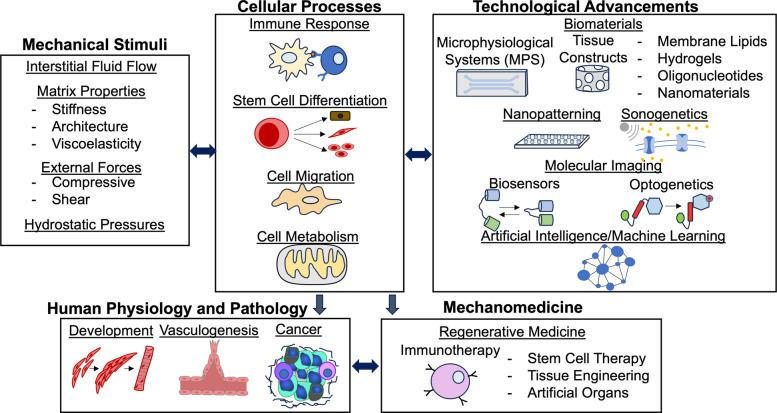
Schematic overview of the focused areas in this perspective

To understand mechanobiological processes, scientists have developed and employed technologies to model, dissect, and control cell functions, which has potential for clinical applications. Additionally, artificial intelligence (AI) and machine learning has been leveraged to help expand our understanding of mechanobiology and mechanomedicine [5]. We met to discuss recent advances and ongoing challenges in mechanobiology and mechanomedicine at the 2025 Cellular and Molecular Bioengineering (CMBE) conference in San Diego, CA (Fig. 1). Following the themes and discussions at the CMBE conference, this perspective article discusses our understanding of mechanobiology in immunoengineering, cell migration, cell metabolism, and stem cell differentiation associated with our understanding of development, vasculogenesis and cancer. We also discuss important technological advancements – including biomaterials, microphysiological systems, nanopatterning, sonogenetics, optogenetics, imaging technologies, AI/machine learning – that are applicable to mechanomedicine, particularly in the context of regenerative medicine and immunotherapy. In closing, we discuss current knowledge gaps, provide our perspectives for ways to address ongoing challenges, and emphasize the urgent need to address current challenges.

### Mechano-immunoengineering

Mechanical cues play an important role in regulating immune cell behavior, from molecular signaling to tissue-scale responses. The field of mechano-immunoengineering leverages the understanding of the mechanobiology of immune cells to design tools for understanding and controlling immunity with spatial and temporal precision. Here we first examine how nanoscale ligand organization—using techniques like nanopatterning and DNA origami—regulates receptor clustering and immune signaling, offering insights for molecular-level immunomodulation. Secondly, we highlight the development of mechano-tunable biomaterials, including artificial antigen-presenting cells (aAPCs) and scaffolds, that use stiffness, viscoelasticity, and ligand presentation to guide T cell activation and expansion. These platforms offer scalable, controllable alternatives to natural APCs for immunotherapy. Finally, we explore sonogenetics, an emerging strategy to remotely program engineered immune cells using focused ultrasound. This approach enables noninvasive, localized activation of gene circuits and immune responses in vivo. Together, these advances define a powerful convergence of mechanics, materials, and synthetic biology for next-generation immunotherapies.

#### Molecular mechanobiology of immune cells

In mechanobiology studies spanning from cellular down to the molecular level, receptor clustering is an important universal phenomenon and mechanism that triggers mechanotransduction and various receptor signaling. For example, integrins form nanoclusters with a diameter of ~ 100 nm in nascent adhesions based on mechanotransduction from extracellular matrix (ECM) [6, 7]. T cell receptors (TCRs) form nanoclusters with a diameter of ~ 200 nm at the interfaces with APCs [8, 9], together with multitype costimulatory, inhibitory, and adhesion molecules organized into a stereotypic geometric structure termed as the immunological synapses (IS) [10]. Advancing from mechanobiology to mechanomedicine, it is crucial to probe cellular and biomolecular interactions at the nanoscale and molecular level, investigate the underlying mechanisms of receptor clustering and signaling, and determine the optimal ligand presentation and arrangement to manipulate cellular functions, which can be used to guide the rational design of nanomaterial-based therapeutics and nanomedicine for optimized outcomes [11–13]. For immunoengineering, multivalent antibody-conjugated nanoparticles have been used as bispecific T cell engager (BiTE) to redirect T cells to tumor cells, which not only provide higher binding affinity and stimulation efficacy than monovalent antibodies, but also a simpler approach at lower cost than constructing multivalent antibody by protein engineering [13]. Dual complementary liposome with tunable ratio of multitype ligands exhibited increased binding to tumor cells, compared to single-targeting liposomes [14]. However, multivalent or multitype ligands are freely distributed on the nanoparticle surfaces without spatial control beyond their molecular density and ratio, to engineer optimal therapeutic outcomes.

**Fig. 2:**
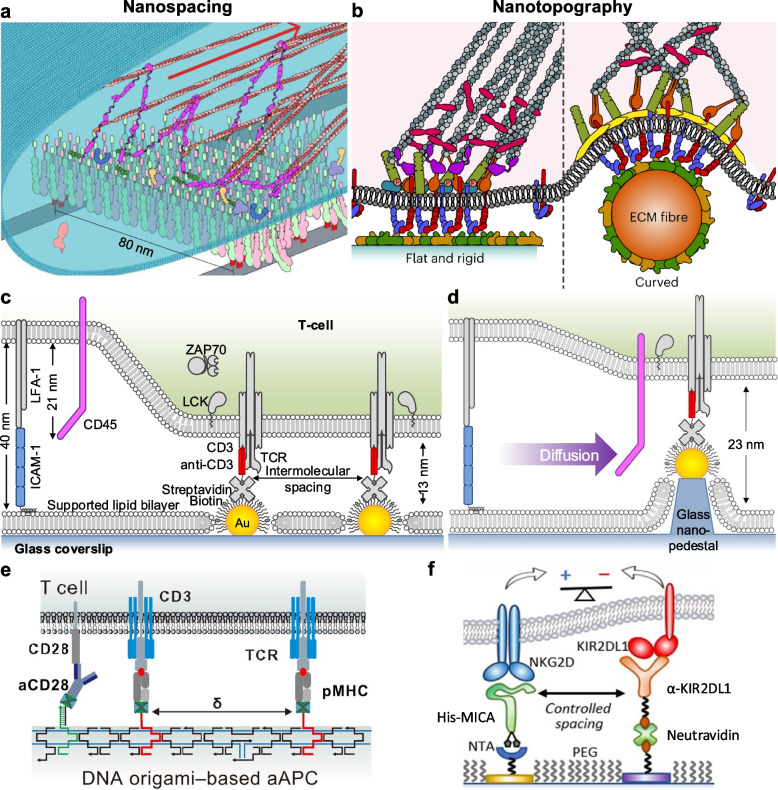
Molecular mechanobiology and mechano-immunology using nanopatterning technologies: ligand spatial control in 2D (lateral nanospacing) and 3D (nanotopography considering the axial direction). **a** FAs on fiber-mimetic 2D nanoline surfaces [6]. **b** Curved adhesions on 3D topographic surfaces [16]. **c** T cell activation on aAPC surfaces of nanoparticle arrays. **d** Particle-tipped nanopedestal arrays with axial elevation of ligand position [9]. **e** DNA origami-based aAPC [25]. **f** Heteromolecular spatial control based on bimetallic arrays [29]

Borrowing the lithographic technology from semiconductor industry (e.g., electron beam lithography), nanopatterning of biomolecules provides biomimetic surfaces to probe and control cellular responses with systematically varied geometric arrangements, enabling unprecedented resolution and precision [15]. Nanopatterns functionalized with ECM proteins have been developed to mimic ECM fibers at different scales. To mimic the in vivo small ECM fibers (diameter 5–20 nm), nanolines with a width of 10 nm were created as one-dimensional (1D) single-molecule binding sites for integrins (integrin head dimension ~ 8 nm), in various two-dimensional (2D) arrangements on glass substrates. It was found that the optimal nanospacing is 80 nm between adjacent fibers for maximal cell adhesion area and total focal adhesion (FA) area ratio per cell (Fig. 2a) [6]. Unlike the abundant FAs on rigid 2D surfaces, curved adhesions are more common in soft three-dimensional (3D) environments, which closely resemble in vivo physiological conditions [16]. Mediated by both integrin and curvature-sensing protein, these curved adhesions are regulated by the membrane curvatures imposed by the nanoscale topography of ECM fibers (Fig. 2b) [16]. Using glass nanopillars and nanobars, it was found that actin fibers form in a curvature-dependent manner with an upper limit of the curvature diameter at ~ 400 nm [17]. The optimal 2D ligand presentation for integrin adhesion is to geometrically match the intrinsic integrin cluster dimension [18], which is determined by factors such as the cytoplasmic adhesion protein complex structures [6] and cell membrane properties. The geometric effect of 3D adhesion is determined by a distinct subset of adhesion proteins, as well as curvature-sensing proteins which recognize topography-induced curvature of a limited range [17]. Since the disruption of cell-ECM adhesions inhibits the migration of certain cancer cell lines, the findings on nanopatterns provide potential therapeutic targets.

For immunology studies, nanoparticle arrays functionalized with TCR binding ligands (e.g., anti-CD3, pMHC) revealed the geometric underpinnings of T cell activation [9]. TCR signaling (e.g., phosphotyrosine, ZAP70) increases gradually with decreasing ligand nanospacing on flat 2D surfaces (Fig. 2c). The inter-membrane spacing generated by TCR-pMHC is 13 nm [19], as mimicked by the nanopattern platform, while the transmembrane tyrosine phosphatase CD45 molecule has an extracellular domain of ~ 20 nm [20]. As described by the kinetic segregation model [20], the close membrane apposition induced by TCR binding excludes CD45 molecules [21]. The CD45 exclusion shifts the local kinase-phosphatase balance to favor TCR triggering [22]. To achieve 3D control of ligand positioning, the nanoparticles can be elevated above the surface by etching the underlying substrate and forming nanopedestals, which in effect increases the inter-membrane axial distance with rigid spacers without incurring entropic penalties associated with flexible spacers inherent to protein engineering (Fig. 2d). A 10 nm axial elevation of binding ligands allows the large transmembrane tyrosine phosphatase CD45 to diffuse into the TCR clusters, which diminishes the signaling, resulting in a sharp lateral spacing threshold at 40 nm. These results directly validate the kinetic segregation model [20]. More importantly, since the TCR cytoplasmic domains have an average length of ~ 20 nm, which reach a close-packed state at a lateral spacing threshold of 40 nm, increasing the potential for T cell activation even if CD45 molecules are not fully excluded [9]. Therefore, a sharp lateral spacing threshold provides translational implications to improve immunotherapy safety and selectivity targeting high antigen expression on tumors versus on normal tissues at lower levels.

Despite above accomplishments, nanopatterning based on top-down lithography is typically bound to surfaces for in vitro cell assays, with limited throughput for clinical-grade manufacturing. Moving forward to in vivo mechanomedicine, it is crucial to develop isolated nanostructures. Using short oligonucleotide staples, a long strand of DNA can be folded to create nanoscale shapes, known as “DNA origami” [23]. By designing the DNA sequences, it is possible not only to program the origami shapes, but also to arrange ligands with nanospacing on the origami templates (Fig. 2e). DNA origami have been used as an alternative to top-down nanopatterning in molecular mechanobiology studies, providing better reproducibility and scalability [24]. More importantly, solution-based DNA origami are more suitable for in vivo translational applications, e.g., molecular vaccines for cancer immunotherapy, which precisely present antigens and ligands for T cell activation and cancer cytotoxicity [25, 26]. A critical challenge is to improve the biological stability in culture medium and in vivo environments. For example, oligolysine-PEG coating has been used to shield DNA origami from low-salt denaturation and nuclease degradation [27].

In the meantime, there is still plenty of room to further explore top-down nanopatterning. For example, existing nanopatterns have been limited to metallic materials (Au) that plasmonically quench fluorescence and, thus, are incompatible with super-resolution fluorescence microscopy. A recent progress of dielectric (TiO_2_) nanopatterning eliminates the plasmonic quenching effect, enabling super-resolution imaging and mechanistic dissection of molecular-scale signaling events in conjunction with nanoscale geometric manipulation [18, 28]. Extending the material selection and functionalization scheme also enables the creation of heteromolecular arrays by placing multitype ligands on different materials (e.g., Au, TiO_2_) through orthogonal chemistries. It was found that the optimal inhibitory condition for natural killer cells is a 40 nm spacing between activating and inhibitory ligands, considering their mismatch of inter-membrane axial distances (Fig. 2f) [29]. The studies will provide important implications for novel immunotherapies involving checkpoints or dual activating and inhibitory chimeric antigen receptor (aCAR:iCAR). Nanopatterning of multiple ligand types will require more efficient orthogonal functionalization with minimized crosstalk, as well as theoretical modeling considering cell membrane properties to provide mechanistic insights. Overall, nanopatterning provides a comprehensive toolbox (various geometries of nanoparticles, nanolines, nanopillars; 2D vs. 3D; single vs. multitype ligands) to mimic and manipulate the molecular organization at cell interfaces for mechanobiology studies towards mechanomedicine applications, e.g., to improve the immune cell activation, selectivity, and direct their differentiation organization in mechano-immunology and mechano immunoengineering [30].

#### Mechano-tunable biomaterials for immune cell engineering

Biomaterials have been strategically exploited to modulate immune cells for the immunotherapy of diverse diseases [31–34]. A rapidly advancing frontier involves the development of biomaterials with well-defined mechanical and chemical properties to enable the scalable ex vivo manufacturing of immune cells such as CAR-T cells for cancer and other disease therapies. This strategy offers a controllable and reproducible alternative to conventional antigen-presenting cells (APCs) in adoptive cell therapy. Here, we highlight recent progress in the development of biomaterial-based artificial APCs (aAPCs) and scaffolds that present activation signals to drive T cell activation and expansion, with an emphasis at the intersection of materials engineering, mechanobiology, and immunoengineering.

**Fig. 3:**
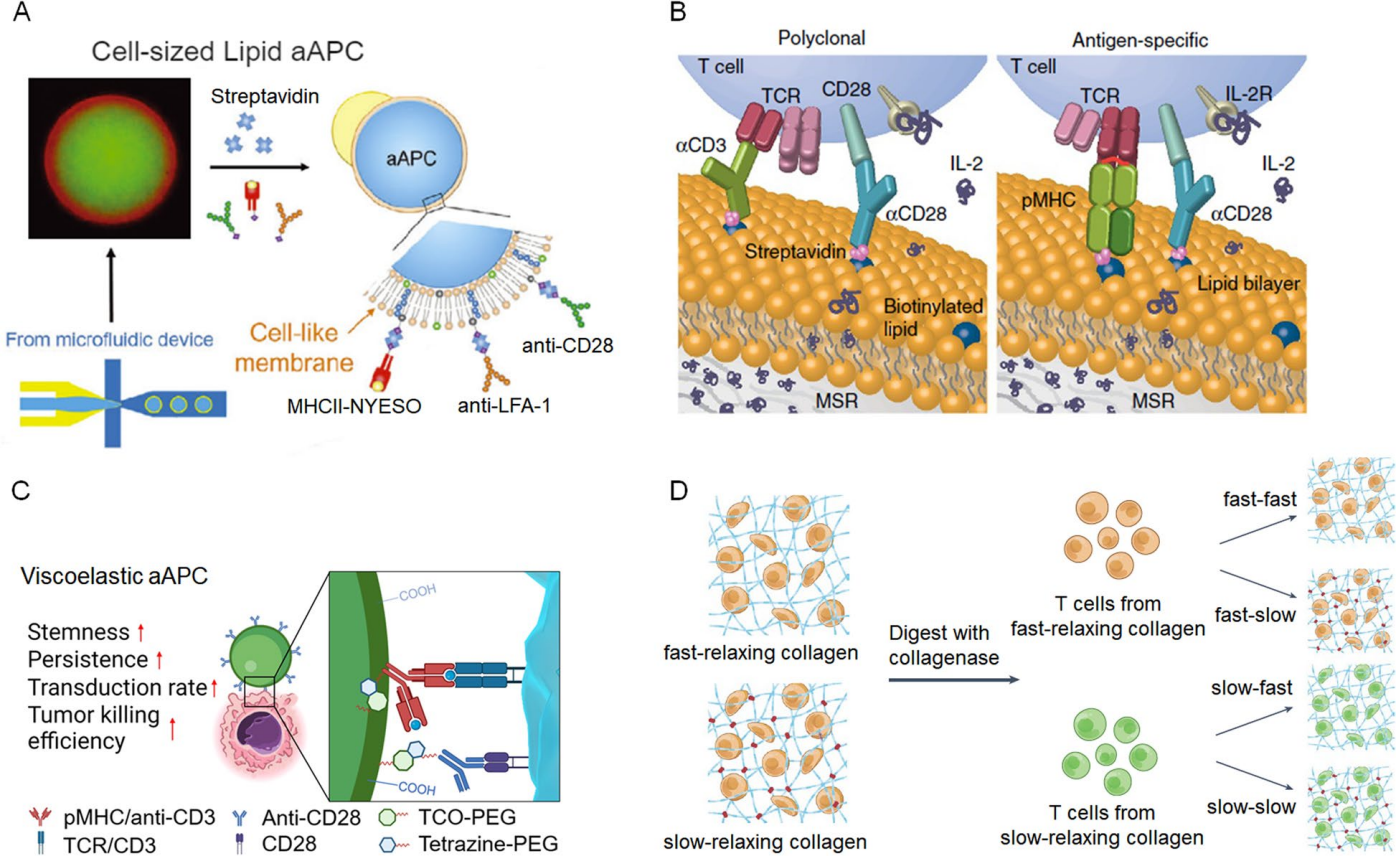
Representative examples of aAPCs and scaffolds for T cell activation. **A** Generation of ASPIRE derived from adenovirus-infected mature dendritic cells; **B** Cell-sized lipid vesicles as aAPCs for antigen-specific T cell activation; **C** Viscoelastic alginate microspheres mimicking the mechanical properties of native APCs; **D** Synthesizing collagen-based ECM mimetic with tunable viscoelastic properties for T cell activation

Cell membrane-based platforms offer considerable advantages by closely mimicking the natural presentation of activation ligands on the surface of antigen-presenting cells [35–40]. Recently, droplet-based microfluidic systems have been developed to fabricate cell-sized lipid vesicles (Fig. 3A), which replicate the mechanical and biochemical properties of native APCs for efficient T cell expansion [41, 42]. Moreover, the shape of the cell membrane- and lipid-coated microparticles has profound effects. For example, biodegradable silica microrods and poly(lactic-co-glycolic acid) (PLGA) ellipsoidal particles coated with lipid bilayers have been used to present T cell-activating signals [41–45]. These anisotropic shapes not only increase resistance to macrophage uptake, but also enhance T cell activation. Compared with Dynabeads, a widely used aAPC, these silica–lipid hybrid aAPCs (Fig. 3B) markedly enhanced primary T cell proliferation, with a distinct CD8^+^ T cell bias attributed to their high aspect ratio. For scalable clinical applications, the inherent instability of lipid materials necessitates strict storage and quality control protocols to preserve their functional stability.

Compared to rigid materials, hydrogels provide superior control over the mechanical presentation of activation molecules. Alginate hydrogels represent a versatile class of biomaterials due to their tunable mechanical properties—achieved through covalent or ionic crosslinking—and their chemical adaptability for biofunctionalization. These features have been harnessed to fabricate aAPCs with controlled size, stiffness, viscoelasticity, and surface activation ligands via microfluidic technologies [46–48]. This platform marked a pivotal shift from conventional, rigid synthetic platforms toward biomimetic systems designed to recapitulate the native biophysical milieu of T cell–APC interactions. Critically, these studies demonstrated that the mechanical properties of aAPCs strongly influence T cell fate. Alginate-based aAPCs, functionalized with anti-CD3 and anti-CD28 antibodies, were shown to optimally induce T memory stem cells (TMSCs)—a population essential for long-term antitumor immunity—when particle diameters were between 5–15 µm and stiffness approximated 20 kPa, consistent with the findings on two-dimensional surface for T cell expansion [49]. In addition, a recent study investigated the effects of aAPC viscoelasticity on CAR-T cell expansion (Fig. 3C) [50], comparing them to commercially available Dynabeads, which exhibit a stiffness of 20–40 MPa but lack viscoelasticity. The superior performance of viscoelastic aAPCs in promoting immune synapse formation, enhancing the CD8^+^/CD4^+^ T cell ratio, expanding CD8^+^ TMSCs, and increasing tumor cell killing capacity strongly supports the emerging paradigm that mechanical cues act as critical co-stimulatory signals for T cell activation and expansion. Furthermore, aAPCs can be used as multifunctional delivery vehicles. The integration of cytokines (IL-2, IL-7, IL-17) and small molecules such as glycogen synthase kinase 3β inhibitors into aAPCs has been shown to further enhance T cell expansion and TMSC induction [46]. In future studies, the translational potential of these aAPCs requires addressing the scalability of microfluidic manufacturing and ensuring consistency in physicochemical properties across large batches.

Recent studies have also addressed the surface functionalization of aAPCs by using a synthetic biology approach. Oligonucleotides, short sequences of DNA or RNA, offer a highly programmable platform for molecular presentation owing to their predictable base-pairing and structural properties [25, 51, 52], which also enable the control of the nanoscale spatial presentation of activating ligands for the construction of highly effective aAPCs. Additionally, inorganic materials such as carbon nanotube–polymer composite has been engineered to present activation signals effectively, promoting T cell expansion for cancer immunotherapy [53]. The composite leverages the unique topography of carbon nanotubes to enhance T cell stimulation and delivers IL-2 locally via polymer-encapsulated nanoparticles. In another approach, a flexible graphene oxide-based antigen-presenting platform was designed to mimic the immunological synapse by anchoring anti-CD3 and anti-CD28 antibodies onto graphene oxide [54], which promotes robust T cell activation and proliferation, enhances CAR gene-engineering efficiency, and supports sustained T cell expansion without the requirement for exogenous IL-2 supplementation.

While aAPCs offer precise molecular control and ease of functionalization, scaffold-based platforms uniquely integrate mechanical and structural cues that mimic the native tissue microenvironment. Scaffolds allow for localized, in situ expansion and can potentially overcome trafficking limitations associated with systemically administered T cells. Unlike aAPCs, which primarily focus on molecular presentation at the surface, scaffold-based systems incorporate biophysical cues from the ECM and biochemical presentations of activation signals to influence T cell function. It is shown that T cells exposed to identical biochemical stimuli can differentiate into functionally distinct subsets depending on the viscoelasticity of their surrounding ECM (Fig. 3G) [31, 55]. Using a norbornene-modified type I collagen ECM model with tunable viscoelasticity—independently controlled from stiffness via bioorthogonal click reactions with tetrazine groups—it was demonstrated that matrix viscoelasticity modulates T cell phenotype and function [31, 55]. These observations mirror tissue-specific gene expression profiles seen in T cells from cancerous and fibrotic tissues, suggesting ECM viscoelasticity as a critical parameter for optimizing T cell products for immunotherapy. In another study, a collagen-crosslinked porous alginate hydrogel was developed to encapsulate T cells and anti-CD3/anti-CD28 antibodies [56]. A similar system is used to deliver T cells and recruit host APCs at tumor resection sites to suppress tumor cell growth [57]. Additionally, hyaluronic acid (HA)-based hydrogels have been engineered as artificial T cell stimulating matrices (aTM) to present the T cell activation signals [58]. The biophysical properties of the aTM—including stimulatory ligand density, matrix stiffness, and incorporation of ECM proteins—were shown to potentiate TCR signaling and modulate the phenotype of both murine and human T cells. Notably, the soft aTM (~ 0.5 kPa) induced most efficient T cell expansion.

Overall, these studies demonstrate that mechanical cues, including shpae of the microparticles, stiffness and viscoelasticity of materials, nanoscale spatial distribution of activation signals, and nanotopography, have significant effects on T cell activation and functions, which can be leveraged for the rational design of aAPCs and scaffolds for more efficient T cell engineering.

#### Sonogenetics for immunoengineering

Mechanobiology and immunoengineering are converging to offer transformative strategies for the spatiotemporal control of cellular functions and provide new ways of immunotherapy. Traditional CAR-T cell therapy has demonstrated striking success in hematologic malignancies [59–61], but its application to solid tumors is limited by life-threatening non-specific targeting of the CAR T cells against normal/nonmalignant tissues (on-target off-tumor toxicities; OTOT) [59, 62–69], T cell exhaustion [70–72] and poor T cell persistence after infusion into patients [73]. To address these limitations, researchers have utilized mechanogenetics, the engineering of cells to respond to mechanical stimuli such as focused ultrasound (FUS), to precisely regulate T cell function in vitro and vivo, creating a new class of immune cells that can be programmed externally with sub-organ precision [74–76].

Initial efforts focused on engineering mechanosensitive ion channels, such as Piezo1, into T cells along with calcium-responsive NFAT-driven gene circuits [77]. In these systems, FUS-generated mechanical stress activates Piezo1, triggering calcium influx and downstream transcriptional activation of CAR genes. This approach demonstrated targeted tumor killing in vivo using Jurkat and primary human T cells. The recently developed CaDox system eliminates the need for microbubbles to activate a gene in cellular system [78]. The CaDox circuit integrates FUS-induced calcium signaling with doxycycline-dependent activation of CD19 transgene expression in tumor cells. SynNotch CAR T cells [79, 80] are then primed by this induced CD19, which triggers expression of a secondary CAR targeting a tumor antigen, enforcing a two-step logic gate. This design restricts T cell cytotoxicity to tumor regions that have been intentionally marked by FUS, reducing off-tumor effects and addressing the challenge of antigen heterogeneity.

In parallel, FUS-induced mechanical vibrations generate heat through frictional losses and viscous damping, leading to localized temperature elevation to trigger gene expression under the control of heat shock promoters [81–83]. MRI-guided FUS can be used to activate CAR expression in T cells at tumor sites, achieving localized antitumor effects and sparing surrounding tissues [84–86]. However, this approach faced challenges in inducing high level of CAR expression and sustaining cell activation following transient stimulation. A major advancement came with the engineer of EchoBack-CAR T cells, which combines an ultrasensitive heat shock promoter identified from a library with a synthetic positive feedback loop linking CAR signaling to its own expression [87]. Upon a single FUS pulse, CAR expression is initiated and subsequently maintained through an antigen engagement, creating a controllable and tumor-constrained immune activation and killing platform. EchoBack-CAR T cells outperformed conventional counterparts in glioblastoma and prostate cancer models, showing durable cytotoxicity, improved T cell persistence, and minimal exhaustion, all while preserving spatial precision.

Collectively, these technologies represent a shift toward "smart" immune cell engineered with external responsiveness, conditional logic, and enhanced safety profiles [75, 76]. Mechanogenetic immunoengineering, particularly via ultrasound-based control platforms, offers a clinically scalable and versatile route to overcome the current barriers in immunotherapy (e.g., CAR-T cell therapy) for solid tumors. Continued development will likely focus on integrating feedback control, minimizing immune clearance, and combining mechanical actuation with molecular targeting strategies to enable next-generation precision immunotherapies.

## Mechanobiology of cell migration

Cell migration is a widespread, fundamental process that is essential for tissue development, tissue repair, and even disease progression. While there are many different modes by which cells migrate, in our perspectives article, we will focus on two: 1. single-cell migration, where cells move as an individual unit, with low correlation to their neighbors, and 2. collective cell migration, where cells move as a cluster, with high correlation to their neighbors. Understanding the mechanics of cell migration has been an active area of research, with prior research generally focused on the contributions of chemical signals to migration. A growing area of research is understanding how mechanical cues impact cell migration and the cellular mechanical responses associated with migration. We have gained some understanding of the mechanical aspects of cell migration including understanding which and how external mechanical cues drive migration, which cell receptors are responsible for sensing and translating mechanical cues, and the downstream signals driving the resultant migration; these topics are the focus of this section.

### Single-cell migration

Traditionally, the mechanisms of single-cell migration have been studied on 2D surfaces, which are rarely representative of the physiological tissue environment [88]. In vivo, cells navigate through complex topographies that impose varying degrees of confinement, including micropores ranging from ~ 1 to 20 µm in diameter, and fiber- or channel-like tracks with widths of ~ 3 to 30 µm [89, 90]. It is now well accepted that cells modulate their migration machinery to adapt to 3D confinement. Cell migration on 2D surfaces is mainly controlled by actin polymerization-driven protrusions, myosin II-mediated contraction and integrin-dependent adhesion and de-adhesion [88]. This mode of migration is commonly referred to as mesenchymal migration. In confinement, cells typically switch to an amoeboid/bleb-based mode of migration, marked by either a stable bleb, observed under conditions of high confinement, high contractility and weak adhesion [91–93], or multiple blebs, often observed in cells with high levels of actomyosin contractility migrating through ECM-coated confining microchannels or ECM environments under conditions of limited pericellular proteolysis [94–96]. Plasma membrane blebs are hydrostatic pressure-induced, actin-free spherical bulges that assist cell migration and invasion by shifting the cell’s center of mass forward and fitting into gaps in the ECM [97]. The nucleus, which is the stiffest and largest cell organelle, serves both as a physical barrier to confined cell migration [98, 99] and a key sensor of confinement, regulating cell migration phenotypes. Confining cells to a 5 μm height stretches the nucleus without compromising the nuclear envelope integrity, leading to the release of calcium from internal stores and the cytosolic phospholipase A2- (cPLA2)-dependent production of the signaling lipid arachidonic acid. In turn, calcium and arachidonic acid elevate cortical actomyosin contractility, promoting plasma membrane blebbing and facilitating confined migration [100]. Under extreme confinement conditions (cell height = 3 μm), the calcium–cPLA2 pathway does not appear to play a dominant role in regulating the migration phenotype [101, 102]. Such conditions induce transient nuclear envelope ruptures, enabling the exchange of material between the nucleus and cytoplasm [103–108]. In adhesive environments, confinement-induced nuclear envelope rupture releases the predominantly nuclear cytokinesis proteins anillin and Ect2 into the cytoplasm, enhancing their cytoplasmic pool [102]. While the Rho guanine nucleotide exchange factor (GEF) Ect2 is primarily diffusely distributed in the cytoplasm, promoting RhoA activation, the scaffolding protein anillin is recruited, upon nuclear confinement, to actomyosin-rich regions at the plasma membrane, particularly at the front and rear edges of cells [102]. There anillin binds to active RhoA, facilitating local activation of actomyosin contractility and promoting bleb-based confined migration and extravasation [102]. Additional mechanisms that drive the transition from mesenchymal to amoeboid migration under extreme adhesive confinement include myosin II–dependent nuclear stiffening [96], which leads to RhoA hyperactivation, and calcium influx mediated by the mechanosensitive ion channel (MIC), Piezo1, which promotes INF2-dependent de-adhesion [101]. Further work is needed to elucidate the crosstalk between nuclear and MIC signaling in the regulation of migration phenotype under varying degrees of confinement. Moreover, less is known about role of nucleus in non-adhesive extreme confinement, where cells typically exhibit a large stable bleb at the front [91–93]. Although studies indicate that nuclear presence within the bleb correlates with enhanced amoeboid migration and that nuclear mechanics influence the transition to the leader bleb phenotype [109, 110], the underlying mechanisms remain unclear.

Blebbing cells display polarized distribution of ion channels, ion transporters and aquaporins, which drive osmotic engine model (OEM)-based confined migration by inducing swelling and shrinkage of the cell front and rear, respectively [111, 112]. At the trailing edge of confined cells, the chloride channel SWELL1 (LRRC8A) mediates regulatory volume decrease, while the ion transporter sodium-hydrogen exchanger 1 (NHE1) accumulates at the front to trigger regulatory volume increase [111, 112]. Simultaneous knockdown of NHE1 and SWELL1 leads to a more pronounced inhibition of confined cancer cell migration compared to either knockdown alone and suppresses extravasation and metastasis in vivo, demonstrating the physiological relevance of the OEM [112]. The cooperative effects among the OEM machinery, the cytoskeleton and the nucleus on confined migration remain incompletely understood and appear to be influenced by the cell microenvironment. At basal fluid viscosity, comparable to that of water, OEM constituents drive confined breast cancer cell migration even after complete disruption of actin polymerization, while simultaneous inhibition of OEM and F-Actin almost completely halts confined motility [111, 112].In contrast, at elevated fluid viscosities, where cells typically move faster [113–116], OEM is insufficient to support cell migration in the absence of F-actin. Higher fluid viscosity, similar to that of blood or interstitial fluid, triggers the formation of a denser actin network at the leading edge, which enhances NHE1 polarization, leading to cell swelling. NHE1-mediated cell expansion elevates membrane tension, promoting Transient Receptor Potential Cation Vanilloid 4 (TRPV4)-dependent calcium influx, and thus activating RhoA/myosin II contractility, which supports the cell cortex at the front [113]. Cancer cells develop Yes-associated protein (YAP)-dependent memory to viscosity, which promotes efficient extravasation in chick embryos, and lung colonization in mice [113]. Furthermore, in viscoelastic and plastic 3D environments, which are commonly encountered in vivo [117, 118], the nucleus acts as a piston and moves forward to pressurize the cell front, stretching the plasma membrane, activating NHE1 and TRPV4, and driving the influx of sodium and calcium ions [119]. The accumulation of these ions within the protrusion locally elevates the osmotic pressure, driving net fluid flow in the protrusion, ultimately increasing protrusion volume, and opening a migration path in confined environments [119]. How OEM affects migration through confined environments of varying stiffness remains unknown, as most confinement studies to date have relied on PDMS-based microfluidic devices, which are relatively stiff. The use of polyacrylamide-based devices, which retain the capabilities of PDMS devices while offering the added benefit of tunable substrate stiffness [120], will enable the systematic investigation of migration mechanisms in confinement across different mechanical environments.

Cells migrating through fluid-filled confined tissue tracks must push the fluid column ahead of them. The resistance to fluid flow, known as hydraulic resistance, guides cell decision-making strategies and directional choices in confinement. Amoeboid cells, such as leukocytes choose the path of least resistance, by using their front-facing nucleus as a mechanical gauge to probe their environment [121]. In these cells, the nucleus is closely followed by the microtubule-organizing center (MTOC), which functions as a spatially associated directional selector controlling the retraction of cytoplasmic protrusions that remain extended in pores of higher hydraulic resistance [121]. Unlike other amoeboid cells, immature dendritic cells display diminished sensitivity to hydraulic resistance due to their ability to take up extracellular fluid through micropinocytosis [122]. In MDA-MB-231 breast cancer cells, elevated hydraulic pressure triggers Transient Receptor Potential Melastatin 7 (TRPM7)-dependent calcium influx, inducing the formation of a thicker cortical actin meshwork containing high levels of myosin IIA motors, which drives cell entry into lower-resistance channels [123], a process that is also energetically favorable [124]. Because most of these insights come from in vitro studies using PDMS-based microfluidic devices or ECM-based hydrogels, further investigation with intravital microscopy is needed to elucidate how migratory cells of diverse origins make directional choices within the dynamic and heterogeneous in vivo environment*.*

Fluid flow (e.g., blood, transmural and interstitial flow) exerts shear (tangential) and pressure (normal) forces on migrating cells. During the transition from tissue migration to intravasation, cells encounter the high shear stress of blood flow. Modeling this transition using microfluidics has revealed that fibroblast-derived cancer cells, such as HT-1080 fibrosarcoma cells, are less sensitive to shear stress than normal fibroblasts [125]. Fibroblasts have higher levels of TRPM7 relative to HT-1080 cells and TRPM7 overexpression suppresses cancer cell intravasation in vivo [125]. TRPM7 activates RhoA/myosin-II contractility and the calmodulin/IQGAP1/Cdc42 pathway, which coordinate to direct cells away from high shear stress environments [125]. As the pore size within a 3D matrix decreases, the pressure-to-shear drag ratio increases [126]. In tightly confined microchannels, where cells occupy the entire channel cross-sectional area, the total drag force on a cell is due to the pressure drag component [127]. In such environments, a large fraction of cells reverse their direction upon exposure to fluid forces and move toward regions of higher pressure [127]. During fluid force-induced cell reversal, actin polymerizes at the new leading edge, activating NHE1, which promotes cell swelling [127]. Concurrently, calcium and myosin IIA accumulate at the new trailing edge, triggering rear contractions. The rigid nucleus, connected to the cytoskeleton via the linker of nucleoskeleton and cytoskeleton (LINC) complex, facilitates cell reversal by controlling intracellular calcium levels and myosin IIA polarity [127]. Highly metastatic breast cancer cells display reduced sensitivity to fluid forces compared to their non-metastatic counterparts, leading to preferential migration towards lower-pressure regions [127]. These studies suggest that fluid force sensing may limit metastatic spread; however direct in vivo evidence is still lacking.

### Collective cell migration

Collective cell migration is the process when cells move as an entire unit in a coordinated fashion. Some examples of collective migration in vivo include tissue morphogenesis such as branching, tubulogenesis, angiogenesis, and vasculogenesis [128, 129]. Tissue repair mechanisms, such as wound healing, is another example of collective migration. Additionally, collective migration is observed in tumor metastasis in circulating tumor cell clusters, which have up to 100-fold greater metastatic potential than single cells, and these patients have significantly worse overall survival and progression-free survival [130]. During collective migration, cells must communicate with each other. For successful collective migration to happen, cells at the front, termed leader cells, send signals to the follower cells. Even though studies investigating collective cell migration mechanobiology is growing rapidly, there is still so much unknown compared to our strong understanding of single cell migration mechanobiology.

During collective migration, cells can adopt different collective migration phenotypes. Recent work investigating MDA-MB-231 breast cancer cell line clusters demonstrate two unique migration phenotypes: spinning/rotating, or translating/invading behaviors [131]. Mechanistic investigation revealed these different migration modes are associated with extracellular matrix remodeling [131]. Specifically, local remodeling is primarily driven by enzymatic cleavage mediated by matrix-metalloproteinases (MMPs), such as MT1-MMP, whereas global remodeling is mediated by integrin-b1 (ITGb1) [131]. Further, when MDA-MB-231 tumor cell clusters adopt a spinning/rotating morphology, they can form lumens [131], a phenotype that is also characteristic of epithelial morphogenesis models [132]. Epithelial cell clusters can also adopt spinning or translating collective migration phenotypes [133]. For epithelial clusters embedded in 3D collagen matrices, formin Dia1 is required for stable adhesion formation that generates large traction forces and deforms collagen fibrils during initiation of branching morphogenesis [133]. These studies demonstrate Dia1 controls stabilization of cell-collagen adhesions. In other studies of branching morphogenesis, researchers found that cell–cell adhesion marker, P-cadherin (Cdh3), mediates cell extension formation which is a precursor step to branching, and this is mediated by RhoA signaling pathway [132].

In vivo, collectively migrating tumor cell clusters also experience mechanical cues, similar to that of single cells. Studies have been done to understand how fluid flow affects collective migration. Using microphysiological model systems that can generate varying interstitial fluid flow rates, it was revealed that primary tumor clusters or organoids (MMTV-PyMT) can migrate with flow over a period of twelve hours, and leader cells localize at the front edge to drive migration [134]. Knocking out discoidin domain receptor 2 (DDR2), a collagen receptor, prevented tumor organoids from responding to fluid flow [134]. To further probe the role of leader cells as a primary driver of tumor migration, single cell sequencing studies were performed and differentially expressed genes in leader cells were identified [135]. One of the top differentially expressed genes in leader cells compared to follower cells was P-cadherin (Cdh3), and loss-of-function studies demonstrate P-cadherin regulates directional collective migration ability in response to biochemical and mechanical cues; this process is regulated by a chemo-mechanical feedback mechanism between P-cadherin and laminin-332 signaling [135]. Further investigation of leader cell generated traction forces reveal that leaders exert higher traction forces compared to follower cells in MMTV-PyMT tumor organoids [136]. Characterization of FA marker, vinculin, demonstrated that leaders have larger and greater number of vinculin FAs compared to followers [136].

The parameters of the extracellular matrix, including stiffness, viscoelasticity, and architecture are also relevant mechanical parameters that impact collective migration. In a collective migration model of the Xenopus laevis cephalic neural crest (NC), cells in these migrating clusters dynamically decrease their stiffness in response to substrate stiffening which triggers collective migration [137]. The authors demonstrate this cell behavior is mediated by a mechanosensitive pathway involving Piezo1-mediated microtubule deacetylation, and collective migration can be initiated by biochemically decreasing microtubule acetylation [137]. Collective cell clusters increased their migration speeds when cultured on viscoelastic collagen matrices and decreased their migration speeds on crosslinked networks which had decreased viscoelastic properties [138]. Additionally, studies have investigated how matrix architecture, specifically whether aligned or randomly oriented matrix, impacts collective migration [136]. Both epithelial cell clusters, madin-darby canine kidney (MDCK) and epithelial-derived primary breast tumor organoids, respond to matrix architecture by sending out cell protrusions that were in the same direction as the matrix fibers [136, 139]. Moreover, physical confinement promotes the dissociation of leader cells from collective strands by activating RhoA through GEF-H1 and the cytokinesis-regulatory proteins RacGAP1 and Ect2. RhoA activation, in turn, enhances myosin II contractility, leading to the dismantling of E-cadherin cell–cell junctions [140].

Progress has been made in understanding some of the collective and individual cell mechanics within the cluster. Techniques such as 3D tracking of cellular forces, were used to demonstrate mouse intestinal organoids in soft hydrogels have non-monotonic stress distribution within the organoid and these mechanical differences can be used to identify functional compartments [141]. For example, in the intestinal crypt, there is a stem cell compartment that pushes extracellular matrix and folds via apical constriction; there is also a transit amplifying zone that pulls on the extracellular matrix and contributes to elongation through basal constriction [141]. Micropatterning techniques have been used to align and polarize epithelial cells, which promotes directional collective migration, and the cell polarization phenomenon is dependent on adherens junction formation or the microtubule network [142].

The physical cues of the microenvironment have long been recognized as critical regulators of cell migration and invasion. However, many fundamental and translational questions are still unanswered. For instance, how migrating cells integrate and respond to multiple concurrent physical cues (a scenario more representative of in vivo conditions) remains unclear since most studies examine only one or two cues. Advanced microfluidic systems with tunable biophysical, biochemical and topographical features [120], will enable researchers to dissect the relative contributions of distinct microenvironmental stimuli to cell migration and provide new insights into the mechanisms governing this process. While the role of uniformly applied mechanical stimuli in regulating cell motility has been extensively studied, how cells interpret gradients of physical cues is far less understood. Extensive literature has investigated the role of plasma membrane and intracellular molecules in cell mechanosensing. How extracellular cues might modulate, including potentially reduce, this process remains largely unexplored. Confined cell migration can trigger DNA damage and genomic instability but it rarely results in cell death [104–106], suggesting the presence of compensatory mechanisms that enable cells to adapt to prolonged confinement. It has been shown that migratory cells can evade death in long-term confinement by downregulating YAP activity, which in turn suppresses p53-mediated apoptosis [143]. A deeper understanding of mechanoadaptation is needed, particularly regarding the long-term effects of sustained mechanical stress on epigenetic regulation, transcriptional and translational activity, and post-translational modifications. Evidence also indicates that cells can develop mechanical memory [113, 144], yet the drivers and mechanisms underlying this phenomenon are poorly characterized. Another open question is whether diseased migratory cells exhibit altered responses to physical cues compared to their healthy or non-migratory counterparts. If such differences exist, they could be used to predict disease progression and clinical outcomes [145–147]. There is also a need to better understand the complexity of innate cell-to-cell heterogeneity and its impact on migration mechanoresponses and disease progression. Recent studies in murine mammary tumors have shown that disseminating cancer cells exhibit reduced adhesion strength, which decreases their sensitivity to stiffness [145, 148], potentially explaining how they navigate away from the rigid tumor microenvironment. Single-cell technologies could uncover this heterogeneity and inform new therapeutic strategies to target specific subpopulations like metastatic cancer cells.

## Mechano- metabolism

Mechanical forces dictate a wide variety of cellular behaviors and biological responses in both healthy and diseased tissues. Some of these mechanobiological responses are achieved via modulation of cell metabolism through interactions between cell–matrix adhesions, cytoskeletal remodeling, and the distinct metabolic pathways regulating catabolic breakdown of glucose, amino acids, and lipids. Mechano-metabolism is a new and growing area of interest in mechanobiology research with disparate applications aimed at understanding basic physiology and identifying novel mechanobiological pathways that are involved in pathological processes. For an extensive treatment of the mutual interactions between mechanics and metabolism, interested readers are referred to recent comprehensive reviews [149–151]. Here, we will briefly discuss the cellular and molecular principles that allow mechanical stimuli to control metabolic processes, key imaging tools that enable visualization of dynamic and spatially heterogeneous metabolic processes, and the mechano-metabolic interactions regulating both single and collective cell migration.

The field of mechanobiology has long established that increasing substrate stiffness results in increased cell proliferation [152, 153]. Yet, the metabolic pathways activated by increased matrix stiffness and the molecular mechanisms mediating such metabolic adaptations are just starting to be investigated. In fact, microenvironmental stiffness is sensed by cells through FAs and cytoskeletal remodeling, which leads to microtubule stabilization and to the generation of actomyosin-rich stress fibers on stiff substrates. Using HeLa cells, Torrino et al. [154] demonstrated that a stiff substrate promotes glutamine uptake. This key amino acid is catabolized into glutamate, which in turn functionalizes the C-terminal tubulin tails via a process known as microtubule glutamylation, ultimately resulting in stable microtubule arrays. Similarly, the metabolic enzyme phosphofructokinase (PFK) is active in human bronchial epithelial cells cultured on stiff substates due to trapping of the PFK-targeting ubiquitin ligase tripartite motif (TRIM)-containing protein 21 (TRIM21), which leads to high rates of glycolysis [155]. Conversely, on soft substrates TRIM21 is free to target PFK, thereby reducing glycolytic while maintaining oxidative phosphorylation (OxPhos). This overall reduction in glucose metabolism is compensated for by an increase in lipid metabolism. In fact, mammary epithelial cells cultured on a soft substrate accumulate sterol regulatory element-binding protein (SREBP) in the Golgi apparatus, causing the release of its cytoplasmic domain, and upregulation of fatty acid and cholesterol biosynthetic enzymes [156]. Therefore, increased matrix stiffness favors the consumption of glucose and glutamine while a reduced matrix stiffness favors the synthesis of fatty acids and cholesterol.

Metabolic changes in cells are commonly assessed via increased transcriptional activity or expression of specific metabolites by means of established genomics, proteomics, and metabolomics technologies [157, 158]. Real-time measurement of metabolism in live cells can be achieved by analyzing metabolic fluxes using the commercial Seahorse Assay [159], although this method results in homogenized measurements across a large number of cells. Spatial and dynamic variations in metabolic activity can be tracked using imaging techniques, which are divided into two main categories. The first category is composed of biosensors, a large family of genetically encoded reporters that have been developed for measuring cell metabolism [160]. These metabolic reporters provide a fluorescent read-out that is proportional to the ATP/ADP ratio (PercevalHR) [161], NAD +/NADH ratio (Peredox) [162], and AMPK activity (AMPKAR2) [163], among others. These reporters can be imaged using standard widefield or confocal microscopy but need to be I) transfected into immortalized cell lines and II) properly calibrated for to account for chemical variations (e.g., pH) that may be interfering with the fluorescent read-out. The second category is represented by label-free methods, primarily Redox and Fluorescence Lifetime Imaging (FLIM). These techniques exploit the fact that metabolic cofactors such as the nicotinamide adenine dinucleotide (NADH) and the flavin adenine dinucleotide (FAD) are auto fluorescent [164]. Therefore, an optical read-out of cellular metabolism can be obtained by measuring their fluorescent intensity (Redox) or their fluorescent lifetime (FLIM). The interpretation of these read-outs can be rather complex but there is a vast literature to rely on [165–168]. Redox data are usually reported as a ratio of NADH-to-FAD intensity [169], while FLIM data are usually reported as a fraction of free-to-bound NADH [170]. In both cases, changes in these ratios have been associated with metabolic shifts towards either Oxphos or glycolysis [171–175]. The key advantage of such label-free methods for mechanobiology studies is that one can perform these measurements on virtually any live cells (immortalized or primary cells) as it relies on the spontaneous fluorescence of naturally occurring molecules. The downside of these methods is that they are complex and require dedicated electronics, often paired with two-photon excitation [176]. Since NADH and FAD exhibit absorption maxima in the blue light spectrum, two-photon microscopy techniques provide two significant advantages for metabolic imaging: they reduce phototoxicity by delivering lower energy during live-cell experiments while simultaneously generating the precise synchronization signal required for FLIM measurements [177].

Mechanically active cells in tissues interact with the surrounding ECM, and such cell-ECM interactions are key to the migratory processes involved with development, wound healing, and cancer invasion. The underlying mechanobiological mechanism, known as mechanoreciprocity, consists of cytoskeletal reorganization to match external biomechanical cues offered by the ECM. Mechanoreciprocity of cell-ECM interactions is a general process that underlies normal tissue homeostasis that is often hijacked by cancer cells to favor the processes of invasion and metastasis [178]. Metabolic plasticity plays a key role in cell migration within complex mechanical microenvironments. Therefore, the concept of metaboreciprocity has been introduced to describe the bidirectional relationship between cellular metabolism and mechanical forces [179]. Metabolism in migrating cells is highly responsive to stimuli from the surrounding ECM microenvironment, yet with few predictable trends [180–184]. In cancer cells, high ECM stiffness has been linked both with elevated rates of glycolysis [155, 185] and OxPhos [180, 182]. For instance, in breast cancer cells, metabolic responses are dependent on cell type: decreasing collagen density increases OxPhos in highly invasive MDA-MB-231 cells while it increases the rate of glycolysis in normal MCF-10A cells [183]. Breast cancer cell invasion has been linked to both glycolysis [186] and OxPhos [187], with inhibition of either metabolic pathway causing significant reductions in migration speed [188]. Single-cell breast cancer invasion in 3D collagen is associated with glycolysis and OxPhos in low and high density collagen, respectively [189].

While mesenchymal cells migrate individually, epithelial cells migrate as a differentiated multicellular collective in which cells expressing basal-like markers (leaders) coordinate the motion of cells expressing luminal-like markers (followers) [190, 191]. Metaboreciprocity in 2D collective cell migration can be examined by measuring and manipulating metabolism in epithelial monolayers migrating on polyacrylamide substrates. The study by Papalazarou et al. [182] showed that ECM stiffness regulates arginine metabolism into creatine biosynthesis in pancreatic cancer cells and that OxPhos supports 2D collective migration. At the same time, DeCamp et al. [192] reported metabolic heterogeneities upon epithelial layer unjamming, the solid-to-liquid transition implicated with collective cellular dynamics [193]. The authors conducted simultaneous measurements of cell dynamics, morphology, mechanics, and metabolism in collectively migrating Madin-Darby Canine Kidney (MDCKII) cells. By combining the metabolic reporter Peredox with label-free FLIM measurements, they found that the leading edge of an MDCKII migrating monolayer undergoes a dynamic shift towards glycolysis while showing enhanced mitochondrial activity. Importantly, the study by DeCamp et al. indicated that metabolic changes preceded changes in cell shape or the onset of cell tractions, thereby suggesting that metabolism plays a causative role in determining the observed pattern of cell migration. Metaboreciprocity in 3D collective cell migration can be examined using 3D spheroids and organoids embedded in fibrous hydrogels, such as collagen. These studies often focus on collective cancer invasion. Extensive work by the Reinhart-King laboratory has demonstrated that collective cancer invasion is implicated with complex patterns of high glucose uptake in leader cells [194] and with a flow of actively proliferating follower cells that require OxPhos [195]. These reports are in agreement with recent spatiotemporal reports of metabolic FLIM within 3D tumor spheroids using both MCF-10A and MDA-MB-231 cell lines [196]. In fact, the work by Karrobi et al. [196] has shown that tumor spheroids display spatial gradients in metabolism, with the core shifting towards a glycolytic metabolism and the leading edge shifting towards OxPhos metabolism. Collectively, these recent studies in both 2D and 3D collective migration support the notion that glycolysis is highly mechanosensitive and plays key roles in proliferation and single-cell migration. At the same time, OxPhos appears to be essential for collective cell migration, even though its contextual role in ECM microenvironments of various stiffness and topography is yet to be explored. Furthermore, the mechanisms underlying mechanical interactions and mechano-metabolic crosstalk between leader and follower cells are yet to be elucidated.

The field of mechano-metabolism is rapidly on the rise and promises to deliver important discoveries in both basic and translational research [197]. In terms of basic mechanobiology, an integrated understanding of mechanotransduction and metabolism will offer novel mechanistic insights into how microenvironmental forces control cell fate [152, 153]. In terms of translational mechanomedicine, unraveling a targetable link between tissue mechanics and cell metabolism will provide much-needed knowledge on the progression of various diseases, thereby offering new opportunities for both diagnosis and treatment. To achieve these goals, an important basic research avenue involves elucidating the mechanisms associated with mitochondrial mechanotransduction and how physical forces dictate mitochondrial dynamics, including mitochondrial fission and fusion [198, 199]. In addition, most of the existing literature has explored how microenvironmental mechanics influences cell metabolism [154–156, 180–184]. However, the links between mechanics and metabolism are likely bidirectional with mechanically regulated changes in metabolism leading to changes in cytoskeletal organization and contractility as well as matrix remodeling via secretion of proteolytic and cross-linking enzymes. From this standpoint, a systematic understanding of the feedback and feedforward links between mechanics and metabolism will be achieved via modulation of key mechanosensitive pathways, such as YAP/TAZ signaling, which have been shown to modulate mechano-metabolic interactions [185]. Finally, the mutual role of mechanics and metabolism in single and collective cell migration appears to be highly contextual and to depend heavily on the specific model system and substrate type. Therefore, we suggest that future work should incorporate multiple substrate types – including confining microchannels, tunable synthetic hydrogels, as well as fibrous natural hydrogels – to analyze systematically metabolic responses to different microenvironments and how inhibition of specific metabolic pathways modulates migratory outcomes [200]. Furthermore, the mechanisms underlying mechanical interactions and mechano-metabolic crosstalk between leader and follower cells deserve to be elucidated in order to gain a better understanding of the process of collective cancer invasion.

## Stem cell mechanobiology and Microphysiological Systems (MPS)

### Stem cell differentiation and mechanobiology

The orchestration of tissue morphogenesis and lineage specification is governed not only by biochemical cues but also by the mechanical and spatial properties of the cellular microenvironment. Although classical differentiation protocols have prioritized soluble factor signaling, mounting evidence underscores the pivotal role of mechanical forces in directing stem cell fate decisions, spatial organization, and morphogenetic outcomes. A wide spectrum of engineered mechanical, including micropatterned substrates, engineered hydrogel scaffolds, and externally applied forces (i.e., dynamically tunable hydrogel systems), serve as instructive cues that influence cytoskeletal organization, polarity, and transcriptional programs. Recent advances in bioengineering have enabled precise manipulation of these parameters, allowing researchers to dissect how stem cells decode and respond to physical signals during development and regeneration.

Micropatterning techniques, such as microcontact printing and photolithography, provide a platform to impose spatial constraints that mimic early embryonic architectures, thereby directing symmetry-breaking events central to germ layer specification [201]. When confined within defined geometries, human pluripotent stem cells (hPSCs) exhibit robust, spatially organized differentiation patterns, recapitulating features of gastrulation and primitive streak formation. This principle extends to three-dimensional models of development, including neural tube organoids, in which imposed tissue domains via micropatterning, following Matrigel application, instructs apical-basal polarity and lumen formation through deterministic, geometry-driven self-organization [202]. Beyond spatial constraint, the mechanical composition of the extracellular matrix plays a decisive role in regulating cell fate. For example, tuning the viscoelasticity of PDMS substrates has been shown to bias mesodermal differentiation trajectories, ultimately enhancing downstream endothelial commitment in iPSC-derived populations [203]. These findings point to a broader principle that the mechanical landscape is not a passive substrate but an active regulator of stem cell identity.

### Engineering perfusable vascular networks in tissue constructs using microphysiological systems (MPS)

A key component to mechanobiology research is access to appropriate model systems that can faithfully replicate mechanical structures and cues of tissues and organs. MPS aims to fill this need by culturing cells in a dynamic microenvironment emulating crucial essential physiological elements for normal function or the pathophysiological condition. Recently, a MPS that models the tumor-vascular interface was design to quantify NK cell migration, infiltration and cytotoxic activity [204]. The MPS model captured the dynamic and spatially confined conditions of the tumor microenvironment to more accurately understand how subsets of NK cells contribute to tissue-specific immune response [204].

A variety of strategies have been employed to incorporate perfusable vascular networks into engineered tissue constructs, including needle molding, soft lithography, and 3D printing-based approaches. MPS provide a powerful platform for promoting heterotypic cellular interactions that enhance parenchymal cell function. For example, co-culturing human umbilical vein endothelial cells (HUVECs) with normal human dermal fibroblasts (nHDFs) within fibrin matrices in a three-channel microfluidic device enables the self-organization of interconnected, perfusable vascular networks. Moreover, this platform permits spatial control over vascular morphogenesis through the introduction of soluble factor gradients, such as VEGF, leading to localized angiogenic sprouting. This modular system further enables the investigation of drug-induced vascular remodeling and endothelial dysfunction. For instance, nicotine exposure has been shown to modulate inflammatory signaling, elevate reactive oxygen species, and perturb vascular architecture within this setting. In a broader context, MPS can also model complex drug-drug interactions by integrating drug-metabolizing hepatocytes, facilitating assessment of systemic pharmacokinetics and toxicity in a multicellular microenvironment [205].

Alternative vascularization strategies include the use of laser-sintered carbohydrate templates to guide the formation of perfusable channels within natural and synthetic hydrogel scaffolds. When hepatic spheroids are embedded in these constructs and exposed to dynamic perfusion, they exhibit significantly enhanced metabolic activity compared to static cultures, underscoring the essential role of vascularization and heterotypic interactions in maintaining hepatic tissue functionality [206].

### Tunable hydrogels to support vascular network formation

Gelatin-based hydrogels crosslinked with microbial transglutaminase offer tunable viscoelastic properties, enabling modulation of stress-relaxation kinetics to support vascular network assembly both in vitro and in vivo. [207] These hydrogels can be engineered to recapitulate tissue-like mechanical microenvironments, which are known to regulate endothelial cell behavior and morphogenesis. Notably, rapidly relaxing hydrogels enhance endothelial cell spreading and facilitate the formation of lumenized networks, while slower relaxing counterparts restrict cell migration and branching. Additionally, gelatin can be chemically modified to support oxygen modulation independently of polymerization kinetics. Using a formulation of gelatin–ferulic acid and aldehyde-functionalized dextran, combined with laccase-mediated polymerization, researchers have developed a hydrogel platform capable of generating controlled hypoxic niches. This system enables the study of oxygen-dependent endothelial morphogenesis and branching behaviors in a hypoxia-inducible factor (HIF)-independent manner, revealing that acute hypoxia alone can promote vascular complexity without requiring canonical HIF signaling [208].

## Molecular imaging and controlling mechanosensing

**Fig. 4:**
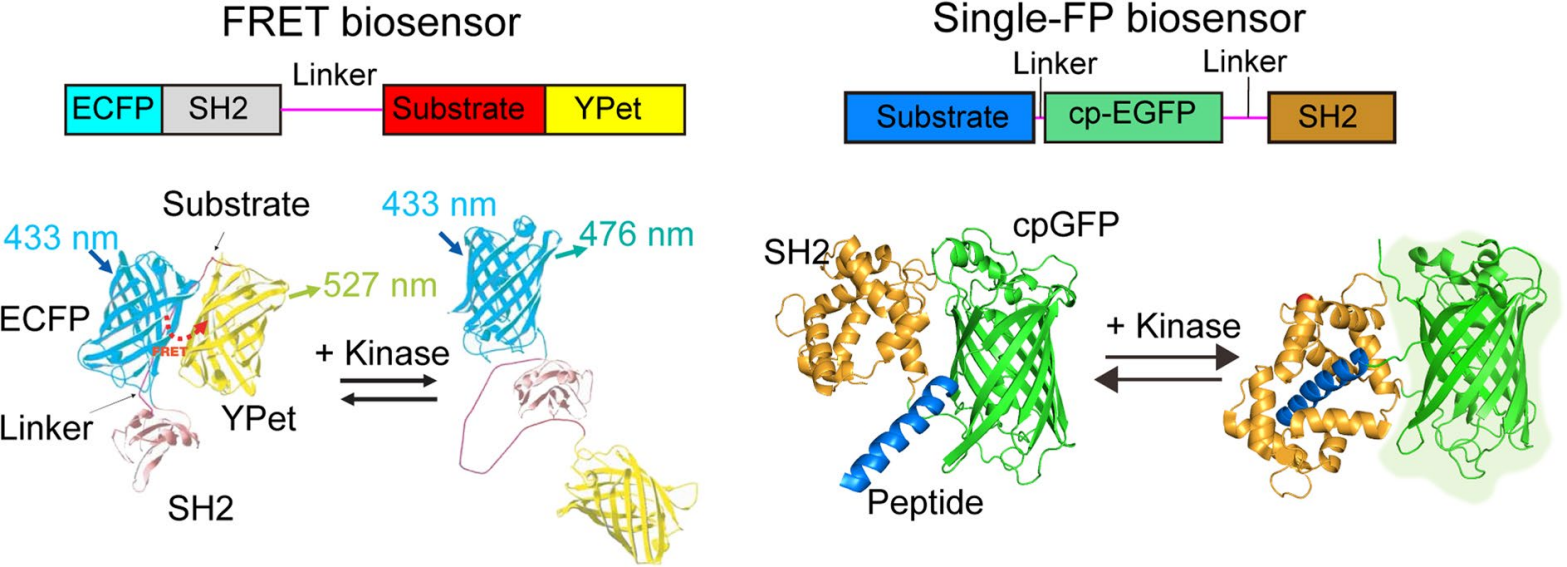
Schematic of FRET sensor (left): ECFP‑substrate‑YPet module switches from a closed to an open, low‑FRET conformation when kinase‑mediated phosphorylation enables intramolecular SH2 binding, shifting emission from 476 to 527 nm. Schematic of Single‑FP sensor (right): A cpGFP flanked by a substrate peptide and SH2 domain brightens upon phosphorylation‑driven docking, giving a single‑channel intensity readout

Biosensors have been widely applied in mechanobiology to monitor force-dependent activation of key signaling molecules, such as focal adhesion kinase (FAK) [209], Src [210, 211], and calcium influx [209] in live cells. One family of biosensors include, fluorescence-based biosensors (i.e. FRET-based and single-fluorophore sensors) have enabled the study of dynamic signaling in response to mechanical stimuli at high spatial and temporal resolution [212]. FRET biosensors consist of a donor and an acceptor fluorescent protein pair flanking a sensing domain that undergoes conformational changes in response to molecular interactions or modifications (Fig. 4). The development of FRET-based biosensors have been particularly valuable in tracking mechanosensitive signaling in response to mechanical forces like substrate stiffness or cell stretching [213]. Advances in high-throughput screening, such as FRET-Seq, have enabled the identification of optimized biosensors with high sensitivity and low background [214, 215], enhancing their utility in future mechanobiology research. Single-fluorophore biosensors, derived from circularly permuted fluorescent proteins or environment-sensitive dyes, offer a simpler alternative with advantages in spectral flexibility and multiplexing [216]. These sensors change their fluorescence intensity or emission wavelength in response to structural changes, making them well-suited for monitoring various biological processes, including mechanotransduction [217]. These tools have been particularly effective in studying cell migration, adhesion, and other force-sensitive processes, revealing how mechanical cues influence intracellular signaling in space and time with high resolutions. Through using FRET biosensors, researchers have been and continue to validate the effects of mechanoreceptors at the molecular levels which can guide new synthetic circuit designs associated with endogenous mechanotransduction pathways. One ongoing limitation of FRET or single fluorophore biosensors is that there is not a clear consensus within the community with regards to how FRET measurements directly correlate to clinical conditions. For example, many researchers have developed FA (i.e. vinculin [218]) and cell junction (i.e. Cadherin-1 [219–221]) FRET tools to measure load at the cell–cell and cell–matrix intersections. While very exciting that cells experience different loads at the cell–cell junctions or cell–matrix interactions depending on the mechanical matrix conditions, it is not entirely clear how to directly translate load measurements to clinical application. Future studies could focus on generating a FRET-based scale that could correlate measurements to human conditions. As mechanogenetic tools continue to develop, these biosensors will play a key role in validating their effects at the molecular level, guiding the design of new synthetic circuits that interact with endogenous mechanotransduction pathways.

While biosensors primarily enable imaging of cellular signals, light-responsive optogenetic systems represent a shift toward actively manipulating cellular processes. These tools are built from part of the family of photoswitchable proteins that can sense light and change conformation upon illumination, allowing a second protein (i.e. the protein of interest) to interact with the light-sensitive protein [222]. Optogenetics are advantageous because it provides a route to control cell signaling pathways using genetically encoded light-sensitive proteins that can perturb intracellular biochemistry at high spatiotemporal resolution. Some of the processes that scientists can control include cell differentiation, migration, signaling, and gene editing [223]. With respect to cell mechanics, using an optogenetic tool to control GEF-RhoA activity in epithelial cells demonstrated increases in traction forces and cell contractility upon optogenetic activation which translocated GEF-RhoA to the cell membrane [224]. Using optogenetics, researchers are elucidating that organelles are also mechanoresponsive: Song et al. developed an optogenetic endoplasmic reticulum (ER)- specific mechanostimulator (LIMER), which regulates calcium (Ca2 +) efflux from ER, inhibits ER-to-golgi trafficking, and increases levels of ER stress markers, binding-immunoglobulin protein (BiP) and phosphorylated eIF2a [225]. In pre-clinical work, optogenetic technologies are being used to activate mechanical nociceptors to increase implant osseointegration [226]. An implantable, wirelessly rechargeable optogenetic stimulation (OS) device was used to stimulate the dorsal root ganglion (DRG) to stimulate Piezo2 + nociceptors as a way to reverse suppression of intracellular mechanotransduction molecule, FAK expression caused by insufficient mechanical stress and promote osteointegration of tibial implants with bone in mice [226]. This study demonstrates the promise of using optogenetics to stimulate mechanotransduction molecules to aid in implant integration. Also, this study demonstrates ideas where optogenetics may be combined with a wireless, implantable device to stimulate the opsins, which could lead to more personalized medicine approaches. As evidenced in this pre-clinical study, an ongoing limitation to using optogenetics for therapeutic applications is the invasiveness and the spatiotemporal range of the biosensor. There are ongoing considerations such as light penetration depth in tissues or how to scale optogenetics from a small animal model to humans; for example, it is not clear what dose or frequency of opsins need to be delivered to reach the necessary number of cells for the optogenetic stimulation to be effective. To address these concerns, researchers are considering developing alternative light-responsive proteins that can be activated by NIR or other wavelengths that can penetrate to deeper depths in tissue. There is high potential for optogenetics to transform patient care, but the success lies in more collaborations between researchers to integrate device technology (i.e. wearable, wireless, and real-time monitoring) with new opsins that can control mechanoresponsive pathways.

## AI/machine learning for mechanobiology and mechanomedicine

With the rapid growth of artificial intelligence (AI) and/or machine learning, more efforts have been done to apply these technologies to increase our understanding of mechanobiology and mechanomedicine [227, 228]. Here we highlight two instances where AI and machine learning have already helped advance our understandings in mechanobiology. First, AI and machine learning tools have been used to generate computational tools that more accurately mimic physiological mechanical properties of tissues with spatial heterogeneity. To accomplish this, researchers used machine learning to train a neural network to identify and predict material parameters using indentation data of biological soft tissues [229]. Second, researchers have begun developing algorithms to more accurately analyze cell response to extracellular matrix mechanical cues derived from large -omics data sets, such as single cell sequencing studies [230]. To do this, researchers used a microfluidic model that can mimic interstitial fluid flow velocities of the breast tumor microenvironment, induced directional invasion, and performed single cell sequencing on primary breast cancer cells [230]. As a first step, an algorithm was developed to separate cells into clusters with more weight regarding biological relevance rather than purely statistical analysis through using data generated from in vitro experiments [230]. This built a strong foundation for downstream single cell data analysis and comparisons. This study is a great example of a first step contributing to our understanding of how mechano-stimuli in physiologically relevant manners can be used to perform large scale -omics studies. In future work, AI and machine learning tools can be applied in a checks and balances manner to compare analysis from microfluidic models and large-scale -omics analysis of patient samples. This is the first steps towards being able to investigate mechano-stimuli separately and in combinatorial patterns.

Another future effort should focus on understanding the relationships between all the different -omics data sets that are being generated, including genomics, epigenomics, proteomics, and transcriptomics data sets in response to cell mechanical behaviors. A large limitation is that analysis of these data sets are generally performed separately without consideration of how all the -omics data sets are interlinked. Thus, understanding the relationships between all the -omics data sets should be high priority. Even the integration of two -omics data sets, with clear biological relevance, would be a huge step in the field because it will allow researchers to make more direct comparisons between cells, organisms, and the extracellular matrix environment since more parameters can be analyzed simultaneously. Future efforts in AI and machine learning should also be invested to integrate mechanical outcome measures, such as force maps or biosensor imaging, with large -omics datasets profiles altogether so that correlations between mechanical metrics and genetic profiles could be elucidated. In the context of translational applications, these types of correlations has the potential to result in development of less invasive or more accurate diagnostic tools, or even decreased time between diagnostic test to actual diagnosis; any of which will significantly improve patient care.

## Looking ahead

Building on foundational work in mechanobiology, researchers are now moving towards clinical translations. One route is identifying new mechanoresponsive therapeutic approaches. For example, there are many foundational studies establishing mechanical features including interstitial fluid flow and matrix architecture (i.e. stiffness, fiber alignment, pore size, etc.) within the tumor microenvironment. Building on this work, studies also demonstrate that tumor cells adapt to mechanical matrix cues which can impact cell response to therapies. Thus, clinical trials are undergoing to test combination therapies that target the mechanical tumor microenvironment and anticancer drugs [231]. Recent work demonstrates the mechanical environment can also induce immune evasion which is a major barrier to immunotherapy efficacy [232–234]. How this happens is an active area of ongoing research with some initial strategies to overcome these barriers testing ideas to use combination treatment of tumor microenvironment inhibitors and immune checkpoint inhibitors [235, 236]. In addition, the approaches that integrate mechanobiology of immune cells, biomaterials engineering, and synthetic biology, as exemplified in molecular engineering, aAPC development, and sonogenetics, will enable more effective therapies that allow the enhancement of metabolic reprogramming of immune cells and better temporal and spatial control of immune cell activation.

Another application of mechanobiology towards mechanomedicine is in the diagnostic field. Researchers have leveraged studies demonstrating metastatic tumor cells have different mechanical signatures (i.e. stiffness or adhesivity) compared to non-metastatic tumor cells^146^. This has led to goals to generate a comprehensive panel of mechanical signatures in metastatic tumor cells that could be used as biomarkers to predict disease progression. In future work, researchers could consider integrating findings from single cell technologies that demonstrate heterogeneity in cell types within tumor samples and perform studies to determine if these different cell types within a heterogeneous tumor sample also have different mechanical properties (i.e. stiffness or adhesivity), and how these different mechanical properties impact invasion or metastasis. Once clear relationships between mechanical properties and different cell types in patient samples are established, this could motivate physicians to include single-cell mechanophenotyping as another diagnostic tool that may be more accurate than current methods. Additionally, researchers are integrating mechanobiological imaging technologies with artificial intelligence for diagnostic use [237]. For example, biosensors that can detect binding forces or fluid flow forces can be used to convert the body’s physiological changes to amplified signals, and then the data generated can be used by AI for analysis.

Finally, mechanobiology is being leveraged in regenerative medicine or tissue engineering approaches which are beginning to shape the emerging field of mechanobiomaterials. The premise of this emerging field is to leverage relationships between material properties and biological responses to optimize tissue repair and regeneration [238]. For example, designing “smart” biomaterials that can incorporate mechanical features to promote cell differentiation down a specific lineage, or to turn tumor cells back into normal cells through inducing certain mechanical cues. Researchers could also leverage findings from single-cell mechanophenotyping studies to design biomaterials that could sense mechanical needs of individual cells and respond immediately so that the local environment is optimal for each cell type. A very simple example where this may be useful is in cancer therapy: after removing a primary tumor, physicians could leave behind a smart biomaterial such that if any residual tumor cells interact with it, the smart biomaterial can immediately respond by altering its mechanical properties that can change tumor cell signaling to prevent invasion.

In conclusion, the field of mechanobiology and mechanomedicine is rapidly growing and new discoveries have resulted in a lot of potential to significantly impact human health. However, a lot of work must still be done to understand clinical implications as associated with detection, diagnostics, prognosis, or therapeutics. We make a call to the community to tackle these challenges.

## Data Availability

Not applicable.
